# Tissue Engineering of Rat Bladder Using Marrow-Derived Mesenchymal Stem Cells and Bladder Acellular Matrix

**DOI:** 10.1371/journal.pone.0111966

**Published:** 2014-12-01

**Authors:** Daniel L. Coutu, Wally Mahfouz, Oleg Loutochin, Jacques Galipeau, Jacques Corcos

**Affiliations:** 1 Department of Biosystems Science and Engineering, ETH Zürich, Basel, Switzerland; 2 Department of Urology, Jewish General Hospital, McGill University, Montreal, Canada; 3 Department of Urology, Alexandria University, Alexandria, Egypt; 4 Department of Hematology and Medical Oncology, Pediatrics & Medicine, Emory University, Atlanta, Georgia, United States of America; Université de Technologie de Compiègne, France

## Abstract

Bladder replacement or augmentation is required in congenital malformations or following trauma or cancer. The current surgical solution involves enterocystoplasty but is associated with high complication rates. Strategies for bladder tissue engineering are thus actively sought to address this unmet clinical need. Because of the poor efficacy of synthetic polymers, the use of bladder acellular matrix (BAM) has been proposed. Indeed when cellular components are removed from xenogenic or allogeneic bladders, the extracellular matrix scaffold thus obtained can be used alone or in combination with stem cells. In this study, we propose the use of BAM seeded with marrow-derived mesenchymal stem cells (MSCs) for bladder tissue engineering. We optimized a protocol for decellularization of bladder tissue from different species including rat, rabbit and swine. We demonstrate the use of non-ionic detergents followed by nuclease digestion results in efficient decellularization while preserving the extracellular matrix. When MSCs were seeded on acellular matrix scaffold, they remained viable and proliferative while adopting a cellular phenotype consistent with their microenvironment. Upon transplantation in rats after partial cystectomy, MSC-seeded BAM proved superior to unseeded BAM with animals recovering nearly 100% normal bladder capacity for up to six months. Histological analyses also demonstrated increased muscle regeneration.

## Introduction

Various congenital and acquired conditions such as exstrophy, cancer and trauma result in compromised bladder capacity or compliance and require bladder replacement or augmentation. Historically skin, bladder submucosa, omentum, dura, peritoneum, seromuscular grafts, small intestinal submucosa and synthetic grafts have been used for bladder augmentation [1]–[3]. These approaches were limited by mechanical, structural, functional or biocompatibility issues. Currently enterocystoplasty is the most effective surgical solution. It can improves continence but is associated with complications such as metabolic disturbances, urolithiasis, increased mucus production, infections and malignant transformation [4]–[7]. Alternative strategies for tissue engineering of bladder tissue are thus actively sought [8].

Tissue engineering requires cells with a supporting scaffold recapitulating the physiological and mechanical properties of tissues. Scaffolds should be non-toxic, have the same mechanical properties as the tissue of interest, and integrate biochemical and spatial cues replicating the properties of native tissue (adhesive cues, mass transport, surface texture and composition) [9].

For bladder tissue, synthetic polymers such as polylactic/polyglycolic acid, polyethylene, and polyvinyl result in graft failure associated with urinary tract infections, urolithiasis, graft contracture and rejection [10],[11]. As an alternative, the use of bladder acellular matrix(BAM) has been proposed [12]–[14] as it possesses the same ECM composition, mechanical properties and complexity as native tissue. BAM from allogeneic, cadaveric and xenogenic sources can be used due to removal of most antigenic proteins [15]. BAMs have been shown in animal models to induce ingrowth of endogenous uroepithelial cells (UCs), smooth muscle cells (SMCs), endothelial cells, and nerve tissues into the scaffold from adjacent parenchyma and partly improved bladder function after cystoplasty [6],[8]. However, smooth muscle regeneration, neovascularization and innervation of the graft were scarce and disorganized. This might lead to bladder fibrosis and affect long-term bladder function [16]. Isolated SMCs and UCs have also been tested in experimental bladder tissue engineering [8], however it is unclear whether functional cells can be isolated from diseased organs.

More recently, umbilical cord-derived mesenchymal stem cells (MSCs) have been used in combination with BAM for bladder reconstruction in a canine model and shown to be superior to unseeded BAM [17]. However, the authors did not report on the urodynamics of transplanted animals and the study was only short-term. Moreover, umbilical cord-derived MSCs are poorly characterized compared to their marrow-derived counterparts and since they are allogeneic, they could be rejected upon transplantation [18],[19].

We here present our efforts to engineer artificial bladder tissue from a xenogenic source of BAM and marrow-derived MSCs in a rat model. Our data show that MSCs seeded on BAM can survive, proliferate and differentiate. Moreover, animals transplanted with MSC-seeded BAMs recovered normal function and nearly full bladder capacity for the duration of the study (6 months) and histological analyses showed better tissue regeneration as compared to animals transplanted with unseeded BAMs.

## Materials and Methods

### Ethics statement

All procedures were approved by the McGill University Animal Care Committee.

### Animals

72 female Sprague-Dawley rats, 250–300 g (Charles River), were used: six for harvesting MSCs, 22 for harvesting urinary bladders and 44 divided into eight groups (Table 1).

**Table 1:** Number of animals per group used in this study.

Group	Number of rats	Procedure	Follow-up duration before sacrifice	Purpose, to define:
1	6	Sham-control	1 month	The effect of bladder manipulation on normal bladder capacity
2	6	Normal Control	N/A	Normal bladder capacity
3	5	PC	1 month	Reduced bladder capacity
4	5	PC	6 months	Reduced bladder capacity
5	5	PC and implantation of non-seeded BAMs	1 month	The effect of augmentation with non-seeded BAMs
6	5	PC and implantation non-seeded BAMs	6 months	The effect of augmentation with non-seeded BAMs
7	6	PC and implantation of MSCs-seeded BAMs	1 month	The effect of MSCs differentiation and regenerative properties
8	6	PC and implantation of MSCs-seeded BAMs	6 months	The effect of MSCs differentiation and regenerative properties

CMG: cystometrograms; PC: partial cystectomy; UCs: urothelial cells; BAMs: bladder acellular matrix; MSCs: mesenchymal stem cells; N/A: not applicable

### Harvesting of Urinary Bladders

Rat bladders were dissected at the bladder neck level and processed immediately. Porcine bladders were received from slaughterhouse and transported in Ringer solution and processed upon arrival.

### Decellularization of bladders

Bladder segments were decellularized with 1% sodium-dodecyl-sulfate (SDS) or Triton-X in hypotonic Tris-HCl with rotation for 3–6 days. DNaseI digestion was done at 50 U/ml with 5 mM CaCl_2_ and 4.2 mM MgCl_2_ for 24 h. Tissues were washed with PBS for five days.

### Antibodies used

Calponin, pancytokeratins AE1/AE3, collagen IV (Abcam), collagen 1, collagen 2 (Cedarlane), PPARγ, Sox9, Runx2 (Santa Cruz Biotechnology), osteocalcin (AbD Serotec), FGFR3, laminin (Novus Biologicals), CD31, CD44, CD45, CD73, CD105, Mac1 (BD Biosciences), α-SMA, Ki67 (Dako).

### Histology, immunofluorescence and immunohistochemistry

5–10 µm paraffin sections were cut. Hematoxylin and eosin (H&E) and Masson's trichrome stainings were performed. For immunofluorescence, sections were blocked with 10% donkey serum(Vector), stained with primary antibodies overnight, stained with appropriate AlexaFluor-conjugated secondary antibodies(Invitrogen) and mounted in Vectashield(Vector). For immunohistochemistry, sections were additionally blocked with avidin-biotin block(Vector). HRP-conjugated secondary antibodies (Jackson ImmunoResearch) were detected using VectaStain ABC(Vector). Histological and immunohistochemical stainings were evaluated by a trained, blinded pathologist at the Jewish General Hospital(Montreal). Alternatively, vibratome sections or whole mount staining were performed.

### Microscopy

Slides were scanned with a Nanozoomer (Hamamatsu). Confocal microscopy was done on WaveFX/Leica SpinningDisk or Leica TCS SP5 microscope. Image acquisition and analysis was done using Volocity5, Leica LAS AF and Imaris (Bitplane) software. Z-stacks were acquired at 0.5–2 µm intervals. Images shown are 3D or maximum projections of entire datasets.

### MSCs isolation and characterisation

Femurs and tibias were dissected from rats. Marrow was flushed in DMEM(Wisent) with 10% FBS. Adherent MSCs at passage 3–4 (seeding density 5000 cell/cm^2^) were used for assays and transplantations. MSCs were differentiated into adipocytes, osteoblasts, chondrocytes and smooth muscle cells as described [20]. Flow cytometry was performed on a FACS Calibur(BD).

### Seeding of MSCs on BAMs

BAMs from rats were gravity-seeded with MSCs on both sides at 1×10^6^ MSCs/cm^3^ density and allowed to adhere for 1 h. Culture medium was added to cover the entire scaffold and changed after 12 h. Organ culture was performed for seven days.

### Partial cystectomy and implantation of scaffolds

Abdomens of isoflurane-anesthetized rats were opened through a midline laparotomy incision. PC was performed by removing 50% of the bladder (dome and upper half). BAMs were anastomosed with 5-0 polyglycolic absorbable running sutures. Muscles and skin were closed using 3-0 and 2-0 Vicryl sutures, respectively. Buprenorphine (0.01–0.05 mg/kg) was administered before the operation and every 12 h thereafter for two days. Trimethoprim (2.2 mg/kg) was given for three days post-operatively. Animals were warmed throughout the procedure and post-operatively until full recovery.

### Cystometrograms

Insertion of a suprapubic tube was performed on isoflurane-anesthetized rats: bladders were exposed for insertion of a PE-90 polyethylene catheter (Clay-Adams) through a cystostomy in the bladder dome, secured with 4-0 silk purse-string suture. Its distal end was delivered subcutaneously through the back of the neck. CMG was performed on restrained, awakened animals 48 h thereafter. A high-sensitivity pressure transducer(AD Instruments Inc.) was connected via 3-way stopcock to the catheter and a KDS100 infusion pump(Fisher Scientific). Room temperature saline was infused at a rate of 0.1 ml/min. Intravesical pressure was monitored continuously with a PowerLab 4/30 ChartPro(AD Instruments). CMG was conducted until the animals urinated(maximum bladder capacity). Three voiding cycles were recorded per animal.

### Histomorphometry

Quantification of urothelium layer thickness was done using NDPView (Hamamatsu) by measuring thickness of PCK+ signal in ten randomly chosen fields of view for each group. Quantification of smooth muscle regeneration was done using ImageJ (NIH) by calculating the ratio of SMA+ area over total tissue area in four sections for each group.

#### Statistical analysis

Data are presented as means ± SEM. Kruskal-Wallis and Kolmogorov-Smirnov normality tests were performed with GraphPad Prism5.

## Results

### BAMs derivation and characterisation

**Figure 1:**
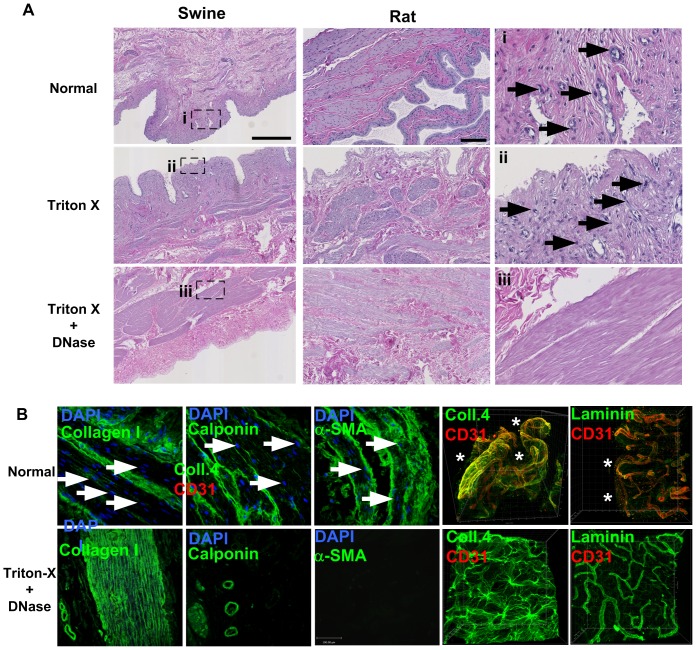
Derivation and characterization of BAMs from multiple species. A) Swine, rabbit and rat bladders were decellularized as described in the methods section. Detergent treatment alone was not sufficient to remove all cellular debris, in particular nuclei, indicated with black arrows (ii). DNase type I was used to remove residual DNA, followed by extensive washes in PBS (iii). Paraffin sections were stained with hematoxylin-eosin (H&E). In all species tested including thick porcine bladders, this protocol resulted in complete removal of cellular components with no obvious damage to extracellular matrix architecture. Scale bars  = 500 µm (swine), 100 µm (rat). B) Confocal microscopy analysis of decellularized rat bladder. In the smooth muscle region of the bladder, the extracellular matrix protein collagen 1 could be detected as long fibrillar proteins that were preserved after decellularizarion. The smooth muscle cell-specific proteins calponin and α-smooth muscle actin (α-SMA, middle and right panels, respectively) were highly expressed in normal bladder tissue but only residual staining could be observed after treatment. DAPI counterstain (white arrows) was also used to confirm complete removal of DNA after treatment. The extracellular matrix protein collagen 4 was observed surrounding and between CD31+ blood vessels in normal bladders and was preserved in BAM. The basement membrane laminin was mainly observed around CD31+ blood vessels in normal bladder but also faintly between them. Although CD31+ endothelial cells were removed in BAM, laminin was preserved sourronding decellularized blood vessels. Asterisks indicate bladder cavity.

Hypotonic solutions and detergents are widely used to derive acellular matrices because they are efficient at lysing cells while preserving the extracellular matrix. In our hands, hypotonic Tris-HCl alone was inefficient at removing cells from bladder tissue. Hypotonic Tris-HCl supplemented with ionic(SDS) and non-ionic(Triton-X) detergents were similarly efficient at removing cells from bladder tissue at the mucosal and adventitial surfaces but cellular debris(mostly nuclei) remained visible deep within the tissue (Figure 1A). Triton-X preserves mucosal glycosaminoglycans and favor cell adhesion better than SDS (less toxic, does not impart a negative charge on the surface, less denaturing to proteins) [6],[13],[21]. Triton-X was thus used in subsequent experiments.

To completely remove DNA, a DNase digestion step was required. This resulted in complete removal of residual DNA (Figure 1A). This approach was successful in all species tested.

We next used immunofluorescence and confocal microscopy to ensure complete removal of cellular debris and preservation of the extracellular matrix (ECM). In sections of normal bladder smooth muscle, most cells co-expressed calponin and α-smooth muscle actin (α-SMA, Figure 1B). After decellularization we could not detect any nuclei in BAMs, even deep within the smooth muscle matrix (Figure 1B, DAPI staining). We also found that the ECM protein collagen 1 was preserved. Conversely, the cellular proteins calponin and α-SMA were nearly undetectable after treatment, although calponin could still be weakly detected in sparse areas. Similarly, the extracellular matrix protein collagen 4 and the basement membrane protein laminin were also preserved in BAM (Figure 1B).

### In vivo characterization of MSCs on BAM

**Figure 2:**
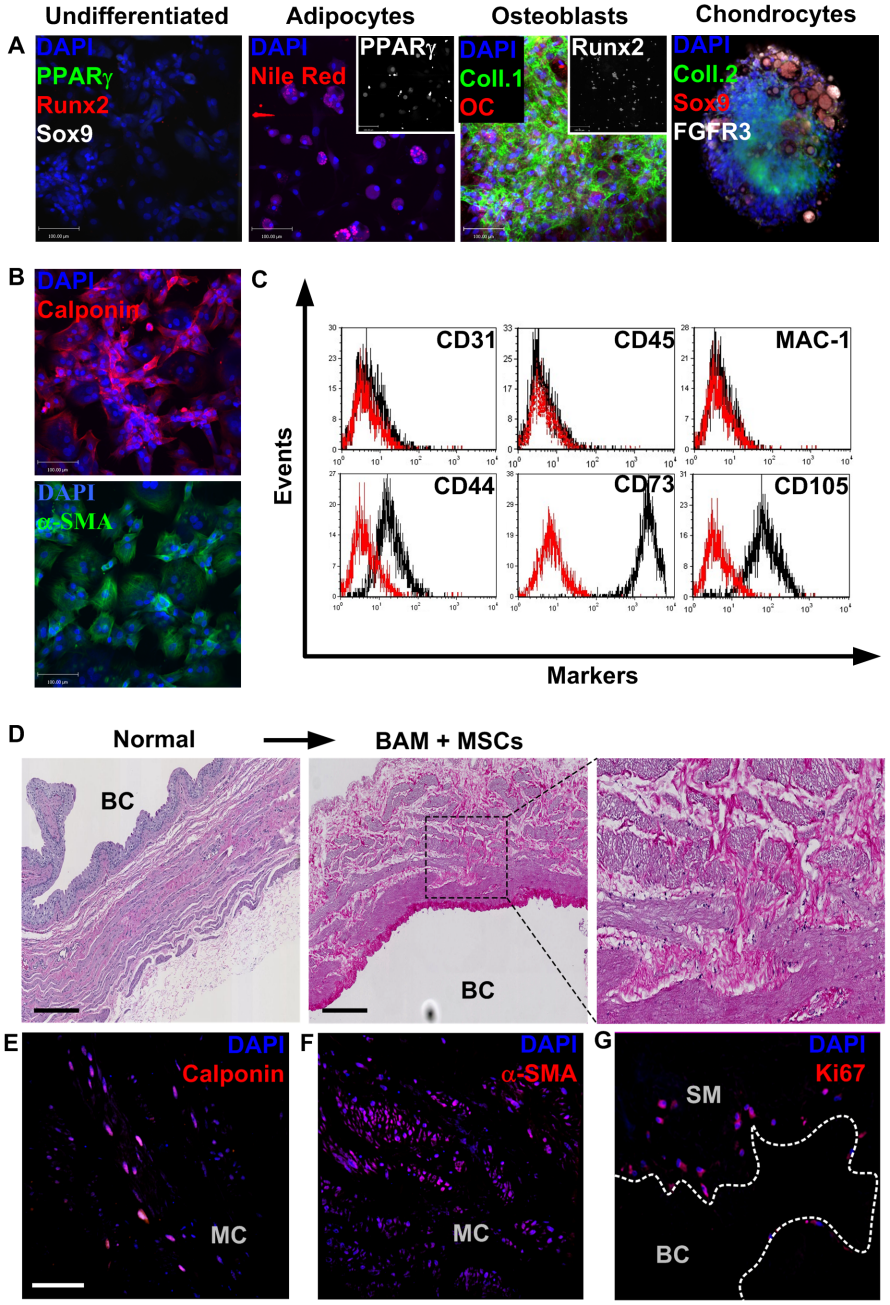
In vitro characterization of rat MSCs alone and on BAM. A) Rat mesenchymal stem cells (MSCs) isolated from bone marrow flushes by plastic adherence. These cells where shown to possess typical MSC plasticity *in vitro*, including the capacity to differentiate into Nile Red/PPARγ positive adipocytes, collagen 1/osteocalcin (OC)/Runx2 positive osteoblasts, and collagen 2/Sox-9/FGFR3 positive chondrocytes. Cells at passage three were used. Scale bars  = 100 µm for all panels. B) Rat MSCs at passage three also possessed the capacity to differentiate into smooth muscle cells in vitro when stimulated with TGF-β, as shown by their upregulation of smooth muscle cell markers calponin and α-SMA. C) Flow cytometry performed on passage four rat MSCs confirmed the cells used in subsequent experiments possessed a typical MSC immunophenotype including expression of SMC markers CD44, CD73 and CD105 and were devoid of endothelial, hematopoietic, and monocytic cells (tested using CD31, CD45 and MAC-1, respectively). D) Bladder tissue was decellularized and seeded with MSCs. After seven days in culture, tissues were formalin-fixed and paraffin-embedded for histological analysis (H&E shown). MSCs were found to adhere to and colonize bladder tissue efficiently, as shown by their broad distribution even deep within tissue. BC: bladder cavity. Scale bar  = 250 µm. E) Rat MSCs found in the middle circular (MC) layer of smooth muscle matrix where found to adopt a smooth muscle cell phenotype, including the expression of the smooth muscle cell-specific protein calponin. Scale bar  = 50 µm. F) Rat MSCs adopt a phenotype specific to their localization within acellular bladder matrix. Cells found within the MC layer of smooth muscle express α-SMA whereas cell attached to the mucosal surface or submucosa remain undifferentiated (not shown). Scale bar  = 100 µm. G) Rat MSCs seeded on BAMs for seven days remain alive and proliferative, as suggested by their expression of the proliferation marker Ki67. Scale bar  = 100 µm.

Undifferentiated MSCs did not express differentiation markers (Figure 2A). Under appropriate conditions they differentiated into Nile Red+PPARγ+adipocytes, Runx2+/collagen 1+/osteocalcin+ osteoblasts, and Sox-9+/collagen 2+/FGFR3+ chondrocytes(Figure 2A). When stimulated with TFG-β, they adopted a SMC-like phenotype evidenced by expression of calponin and α-SMA (Figure 2B). Flow cytometry analysis showed MSCs expressed typical markers including CD44, CD73 and CD105 and were devoid of macrophages, endothelial and hematopoietic markers(MAC-1, CD31 and CD45, respectively)(Figure 2C).

MSCs seeded on BAMs adhered well and invaded deep within the matrix (Figure 2D). We observed more cells in smooth muscle matrix than on mucosal surfaces. This may reflect the endogenous expression by MSCs of adhesion molecules similar to SMCs but not UCs. MSCs in the middle circular layer of smooth muscle expressed calponin (Figure 2E) and α-SMA(Figure 2F), confirming their SMC-like phenotype after culture on BAM. MSCs attached to the mucosal surface or submucosa did not express smooth muscle markers (not shown), suggesting that MSCs fate and phenotype can be instructed by environmental cues from BAM. We confirmed MSCs seeded on BAMs remained viable and proliferative by staining for the proliferation marker Ki67 (Figure 2G).

### Performance of BAMs *in vivo*

We transplanted BAMs alone or MSCs-seeded BAMs in rats after partial cystectomy (PC) and compared them with sham operated animals or animals with PC without augmentation. Cystometrograms (CMGs), histology and immunohistochemistry (IHC) were performed at one and six months post-implantation. BAMs and MSCs-seeded BAMs were generally well tolerated. Four animals in total developed vesical calculi (three in BAM alone group, one in MSCs-seeded BAM group). These were removed during cystostomy prior to CMGs at six months. One animal with calculus in BAM alone group did not survive.

**Figure 3:**
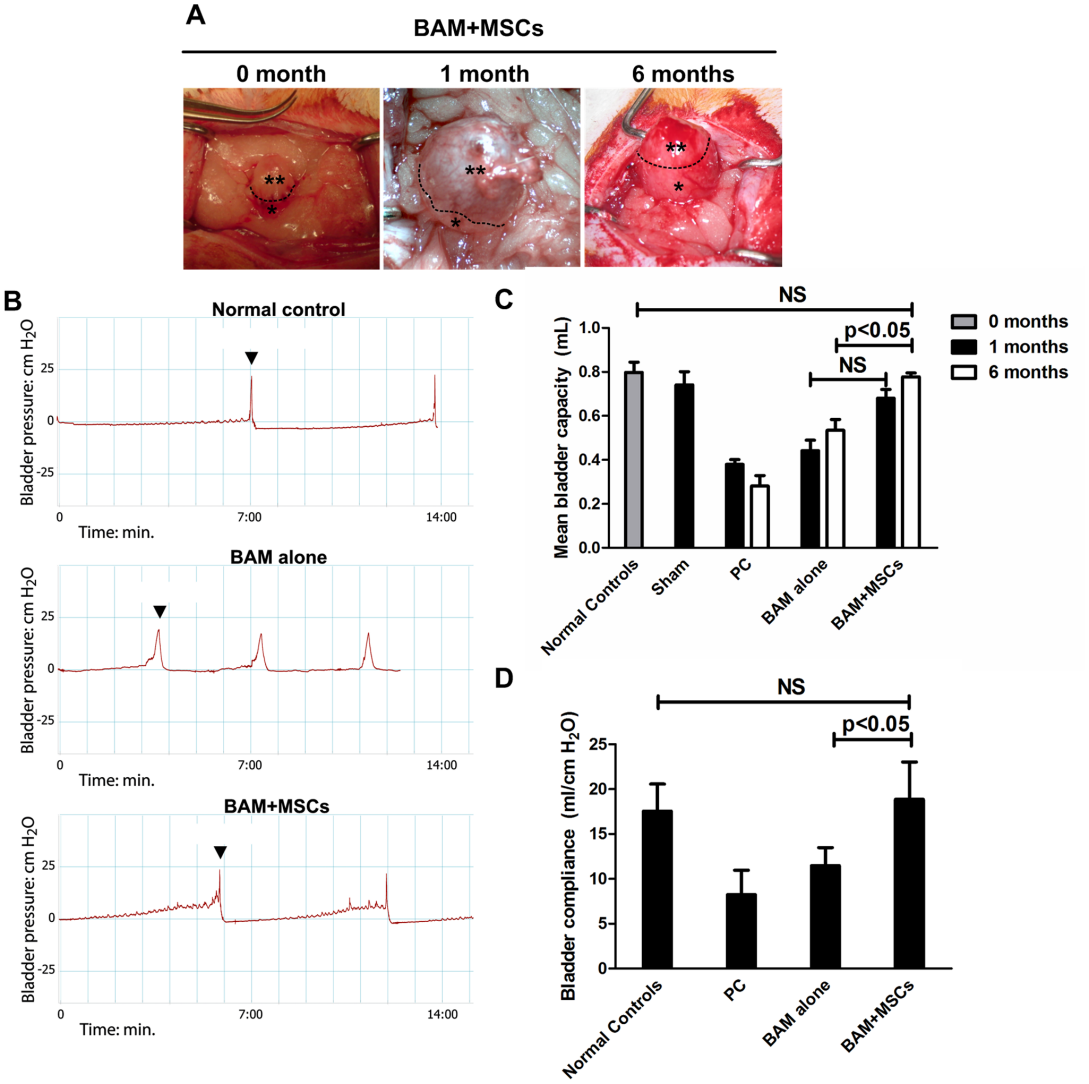
Performance of engineered bladders *in vivo*. A) Gross appearance of MSCs-seeded BAMs immediately after transplantation (0 month) and before retrieval (1 month and 6 months). The anastomosis between native (*) and engineered (**) bladder is indicated by a dashed line. Note that the anastomosis is inconspicuous at 1 and 6 months and that the graft appears normal and well vascularized. B) Representative CMG graphs of animals from normal control group, partial cystectomy (PC) group and MSCs-seeded BAM group. Arrowheads indicate micturition. C) Urodynamic data from all animal tested was plotted and compared using Kruskal-Wallis tests. MSCs-seeded BAMs performed better than unseeded BAMs at 6 months. At 6 months, MSCs-seeded BAMs had restored full bladder capacity when compared to normal controls. NS: not significant. D) Bladder compliance of all animals at 6 months shows that MSC-seeded BAM group outperforms BAM alone group.

MSCs-seeded BAMs appeared normal with no evidence of diverticular formation. Thickness of grafted segments was similar to that of native tissue (Figure 3A). The interface between engineered and native bladders was not conspicuous. Reconstructed bladders were covered by well-vascularized connective tissue at six months post-transplantation (Figure 3A, MSCs-seeded BAMs shown). However, shrinkage of grafts was observed in unseeded BAM group (not shown).

Similar mean bladder capacities (MBC) were recorded in the normal control and sham control groups (0.79±0.12 vs 0.74±0.15 ml). Similar MBCs were also recorded in PC group at one and six months (0.37±0.68 vs 0.28±0.1 ml, approximately 45% normal bladder capacity). The BAM alone group increased their MBCs to 55% and 67% of preoperative values at one and six months, respectively. MSCs-seeded BAMs group reached MBCs of 85% and 97% compared to precystectomy volume at one and six months, respectively. Representative CMGs are shown in Figure 3B. CMG data for all groups are summarized in Figure 3C. All groups followed Gaussian distributions according to Kolmogorov-Smirnov normality tests. At six months, mean bladder capacity and bladder compliance was significantly increased in the MSC-seeded BAM group compared to BAM alone and showed no significant difference from normal controls (Figure 3C–D).

**Figure 4:**
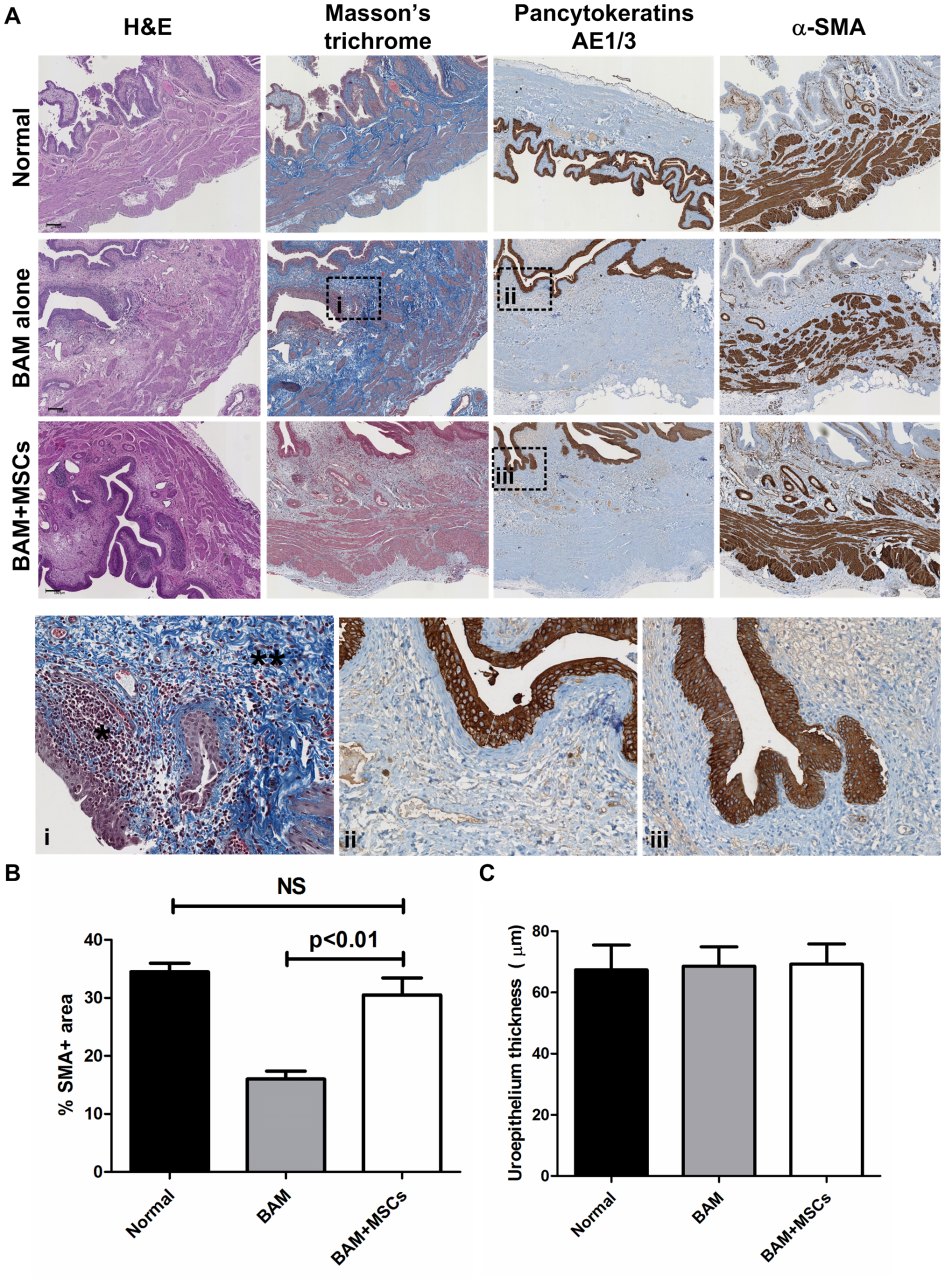
Histological analysis of transplanted engineered bladders. A) Engineered bladders from BAM alone and MSCs-seeded BAMs were retrieved at 1 (not shown) and 6 months (shown) and compared to normal bladders. H&E staining reveals that all bladders possess a tri-layered organization including urothelium, lamina propria and smooth muscle. Masson's trichrome staining shows that unseeded BAMs had more fibrosis (**) and myofibroblastic proliferation (*) compared to normal controls and MSCs-seeded BAMs. IHC for pancytokeratins AE1/3 reveals multilayered urothelium in both unseeded and MSCs-seeded BAMs. IHC for α-SMA shows that smooth muscle fibers in MSCs-seeded BAMs were thicker, more organized and more fascicular than in unseeded BAMs. Scale bars  = 150 µm. B) Histomorphometry of SMA+ area over total tissue volume confirms higher smooth muscle regeneration in MSC-seeded BAM group over BAM alone group at six months post-transplant. C) Urothelium layer thickness was comparable in all groups at six months post-transplant.

All retrieved bladders possessed a tri-layered organization including urothelium, lamina propria and muscularis propria (Figure 4A). In MSCs-seeded BAMs group, organized muscle bundles were present in the graft at one month albeit attenuated in thickness compared to normal controls (not shown). At six months, muscle bundles were more prominent with increased thickness (Figure 4A). Muscle bundles were clearly present in the central part of the grafts. This was further evidenced by immunostaining for α-SMA. In unseeded BAMs group, smooth muscle was present but was disorganized without a fascicular pattern, and with attenuated thickness compared to the MSCs-seeded group. Myofibroblastic proliferation was observed (Figure 4i), suggesting reparative rather than regenerative process. Furthermore, fibrosis and increased collagen deposition were observed. Histomorphometry confirmed that muscle regeneration was higher in MSC-seeded BAM group compared to BAM alone (Figure 4B). Multi-layered urothelial regeneration was equally observed in both groups, as evidenced by staining for pancytokeratin AE1/AE3 (Figure 4ii and iii). Quantification of urothelium layer thickness showed no difference between the groups (Figure 4C).

## Discussion

In the present study, we evaluated a tissue engineered bladder created from BAM seeded with marrow-derived MSCs. As mentioned above, the use of BAM in bladder augmentation strategies has been proposed [14] to circumvent undesirable side effects associated with the current gold standard, enterocystoplasty. Although BAM has been shown to partly improve bladder function in animal models, smooth muscle regeneration, innervation and vascularization were limited. To circumvent these issues, we propose the use of MSCs which can accelerate tissue repair by directly differentiating into smooth muscle cells but also through their paracrine stimulation of angiogenesis and nerve ingrowth and immunomodulatory properties.

MSCs have been tested in combination with small intestinal submucosa in swine [21] and baboon [22] models and were shown to be superior to unseeded scaffold [22]. However, for reasons stated above (mechanical properties, spatial and biochemical cues) it is likely that BAM represents a better supporting scaffold for bladder tissue engineering. Recently, Antoon et al. [23] reported that murine MSCs could be seeded on swine BAM in vitro where they proliferated and differentiated. However, they did not test those constructs in vivo and they did not disclose how the decellularization process was achieved. A report by Burmeister and colleagues [24] suggests that rat bladder is endowed with intrinsic regenerative properties we were unable to observe this in our partial cystectomy group only, even six months post-operation. These discrepancies could be related to the different strains of rats used in both studies or more likely may be explained by the younger age of the animals used in the study by Burmeister et al. Although histological and functional differences in bladder tissue exist between rats and other mammals, our study aimed mainly at testing the *in vivo* viability of engineered bladder tissue made of MSC-seeded BAM and serves as a proof of concept for testing this approach in larger mammals, potentially using xenogeneic bladder tissue.

Marrow-derived MSCs have now been extensively tested in human clinical phase I safety trials for a variety of conditions (over 340 registered clinical trials to date; http://clinicaltrials.gov) and current methods to isolate, expand and transplant them are considered safe [25],[26]. Our study supports these observations in the context of bladder tissue engineering. Indeed, we observed neither signs of malignant transformation of the cells nor undesirable differentiation into bone or cartilage within BAMs. In addition, the immunosuppressive properties of MSCs might overcome immune responses against residual antigenic proteins such as calponin in BAMs, although we observed no inflammation or immune cells infiltrate into neither BAMs alone nor MSCs-seeded BAMs.

Our study demonstrates better smooth muscle regeneration in engineered bladders made with MSC-seeded BAM compared to BAM alone. This resulted in baldder capacity and compliance similar to normal controls six months after transplantation. Our study however did not assess whether MSCs engrafted long term as smooth muscle cells in the engineered bladders or merely accelerated endogenous repair mechanisms through paracrine effects. To assess the fate of transplanted MSCs would have required the use of genetically labeled cells expressing a reporter protein such as GFP, LacZ or luciferase. However, since these xenogenic proteins might be immune-rejected [27],[28],[29] in rodents we decided not to perform fate mapping of MSCs in this study. However, the fact that MSCs differentiated into smooth muscle cells on BAM in vitro and their transplantation resulted in higher muscle regeneration suggests that MSCs did engraft as smooth muscle cells in vivo.

As already mentioned, other groups proposed the use of BAM for engineering of bladder tissue. However, there is no consensus on the best method to achieve efficient decellularization and some studies do not disclose the method used. We here tested several methods and showed that the use of detergents in hypotonic buffer followed by nuclease digestion was highly efficient in removing cellular components. This is in line with other studies showing that this type of treatment efficiently removes cellular components while preserving the extracellular matrix and the mechanical properties of the tissue while allowing seeding of exogenous cells [30],[31]. Also consistent with others [30], we found that some intracellular components (in this case calponin) were still present at low levels in BAM but this did not appear to cause rejection upon transplantation in outbred animals. We observed similar results in BAM derivation using both SDS and Triton-X as detergents. However, SDS has been shown to denature proteins, decrease glycosaminoglycans content, and impede cell adhesion in BAM [6],[13],[21],[32], we propose the use of Triton-X to derive BAM amenable to MSC seeding, adhesion, proliferation and differentiation.

In summary, we demonstrated the in vivo superiority of MSCs-seeded BAMs compared with unseeded BAMs in bladder tissue engineering. Our approach is fully translatable to large animals and humans, where autologous MSCs could be seeded on allogeneic, cadaveric or xenogenic BAMs. The method presented here is a viable alternative to current treatment modalities and should prevent most complications associated with them. This study demonstrates the superiority of MSCs-seeded BAM compared to BAM alone in bladder augmentation and provides a strong basis to test our novel approach in large animal models and eventually in humans.
